# Integrating Radioprotective Agents into Post-Mastectomy Radiotherapy: Optimization of Reconstructive Outcomes in Breast Cancer

**DOI:** 10.26502/jsr.10020395

**Published:** 2024-10-21

**Authors:** Nathan Ramachandran, Nagi Ayoub, Devendra K Agrawal

**Affiliations:** 1 Department of Biology, Union College, Schenectady, New York, USA; 2 Westfield Plastic Surgery Center, Omaha, NE; Creighton University School of Medicine, Omaha, Nebraska, USA; 3 Department of Translational Research, College of Osteopathic Medicine of the Pacific, Western University of Health Sciences, Pomona, California, USA

**Keywords:** Amifostine, Beta-carotene, Breast cancer, Cancer treatment, Glutamine, Manganese superoxide dismutase, N-acetylcysteine, Pentoxifylline, Post-mastectomy radiotherapy, Radioprotection, Radiotherapy, Reconstruction, Tempol, Vitamin E

## Abstract

Surgical intervention utilizing various approaches is a cornerstone in the management of breast cancer. The surgical approaches include lumpectomy, mastectomy, axillary lymph node dissection, and primary or delayed reconstruction. Post-mastectomy radiotherapy is frequently recommended in cases of advanced tumors and extensive lymph node involvement. However, there are several adverse effects of radiotherapy. In this article, we critically reviewed the various complications. Additionally, we discussed the biological basis of radiation-induced tissue damage, the impact of implant-based and autologous tissue reconstruction, and the functional and aesthetic results of the reconstruction. Indeed, several radioprotective agents can attenuate the adverse effects of post-mastectomy radiotherapy while sustaining oncologic efficacy. Radioprotective agents, including free radical scavengers and antioxidants, offer promising strategies to protect tissues from the oxidative stress and inflammation induced by radiotherapy. The role of several radioprotective agents, including amifostine, N-acetylcysteine, tempol, manganese superoxide dismutase (MnSOD) plasmid liposomes, vitamin E, and beta-carotene has been analyzed with a focus on their logistical applications in breast reconstruction. Despite several challenges, the integration of radioprotective agents into post-mastectomy radiotherapy protocols offers significant potential to improve reconstructive outcomes. Development of novel radioprotective agents with improved selectivity and fewer side effects and large-scale clinical trials in diverse group of patients are warranted to determine long-term safety and efficacy.

## Introduction

Breast cancer remains a predominant cause of morbidity and mortality among women today [1]. With over 2.3 million new cases and 660,000 deaths globally in 2022 alone, this disease presents an ongoing burden to individual patients, families, and healthcare systems worldwide [2]. The distribution of this burden is not proportional, however, as less developed countries consistently face much higher mortality rates, largely due to insufficient screening protocols, limited access to diagnostic centers for early detection, and lower healthcare standards altogether [3,4]. Although substantial progress has been made over the years, particularly with the advancement of screening mammography, targeted therapies, and reconstructive surgical techniques, these persistent disparities underscore the urgent need for innovative strategies to address the challenges of breast cancer across diverse global contexts [5].

The treatment of breast cancer is inherently multidisciplinary, encompassing a wide array of modalities designed to address both localized and systemic disease [6–8]. Surgical intervention is a cornerstone of treatment, with various approaches including breast-conserving surgery (lumpectomy), mastectomy, axillary lymph node dissection, and primary or delayed reconstruction [9,10]. The choice of surgical strategy is often guided by tumor characteristics and patient preferences [10]. Concurrently, adjuvant therapies such as radiotherapy, chemotherapy, and hormone therapy are essential in reducing the risk of recurrence and improving oncologic outcomes [11,12].

Radiotherapy, in particular, plays a pivotal role in treatment for patients undergoing breast-conserving surgery, or those at high risk of recurrence following mastectomy [13]. In such cases, post-mastectomy radiotherapy (PMRT) is frequently recommended due to the presence of advanced tumors and extensive lymph node involvement [14,15]. The primary objective of PMRT is to eradicate any residual microscopic disease within the chest wall and adjacent tissues, thereby significantly decreasing the likelihood of locoregional recurrence [16–18]. Evidence supporting the efficacy of PMRT is robust, with numerous studies demonstrating its ability to improve overall survival rates in selected patient populations [17,19]. However, the therapeutic benefits of PMRT are offset by its tendency to adversely affect the surrounding normal tissues, notably those involved in breast reconstruction [20].

Radiotherapy works by damaging the DNA of rapidly dividing cells. Although this mechanism is effective against cancer cells, it can negatively affect healthy cells as well [21]. In the context of breast reconstruction, radiotherapy can induce a variety of complications, which will be discussed in this review. Moreover, as a result of these complications, the functional and aesthetic results of the reconstruction may be compromised, necessitating additional surgical interventions to address these complications [22]. For instance, radiation-induced fibrosis can lead to an irreversible stiffening and hardening of connective tissue, causing a decrease in size and greater distortion of the reconstructed breast [23,24]. Similarly, capsular contracture, characterized by the formation of a thickened capsule around a breast implant, can cause immense pain and deformity [25].

The detrimental impact of radiotherapy on reconstructive outcomes highlights the need for strategies that can mitigate its adverse effects, whilst still preserving its oncologic efficacy. This necessity drives the exploration of radioprotective agents: substances that can protect normal tissues from the harmful, oxidative effects of radiation [26,27]. These agents hold the potential to enhance both functional and surgical outcomes of breast reconstruction, as they can minimize the incidence of complications [28]. Subsequent investigation and integration of these agents into clinical practice holds promise not only for improving surgical outcomes, but also the overall quality of life for breast cancer survivors, a critical focus in modern oncologic care. Given these considerations, this novel review aims to evaluate the integration of radioprotective agents into post-mastectomy radiotherapy protocols, focusing on their mechanisms and potential to optimize reconstructive outcomes. The biological underpinnings of radiation-induced damage, the differential impact on implant-based versus autologous reconstructions, and the clinical implications for functional and aesthetic results will be explored. Through a comprehensive analysis, the review seeks to contribute to the advancement of breast cancer treatment, particularly in the context of post-mastectomy radiotherapy and reconstruction, an area yet to be thoroughly explored by existing literature.

## Biological Basis of Radiation-Induced Tissue Damage

**Figure 1:**
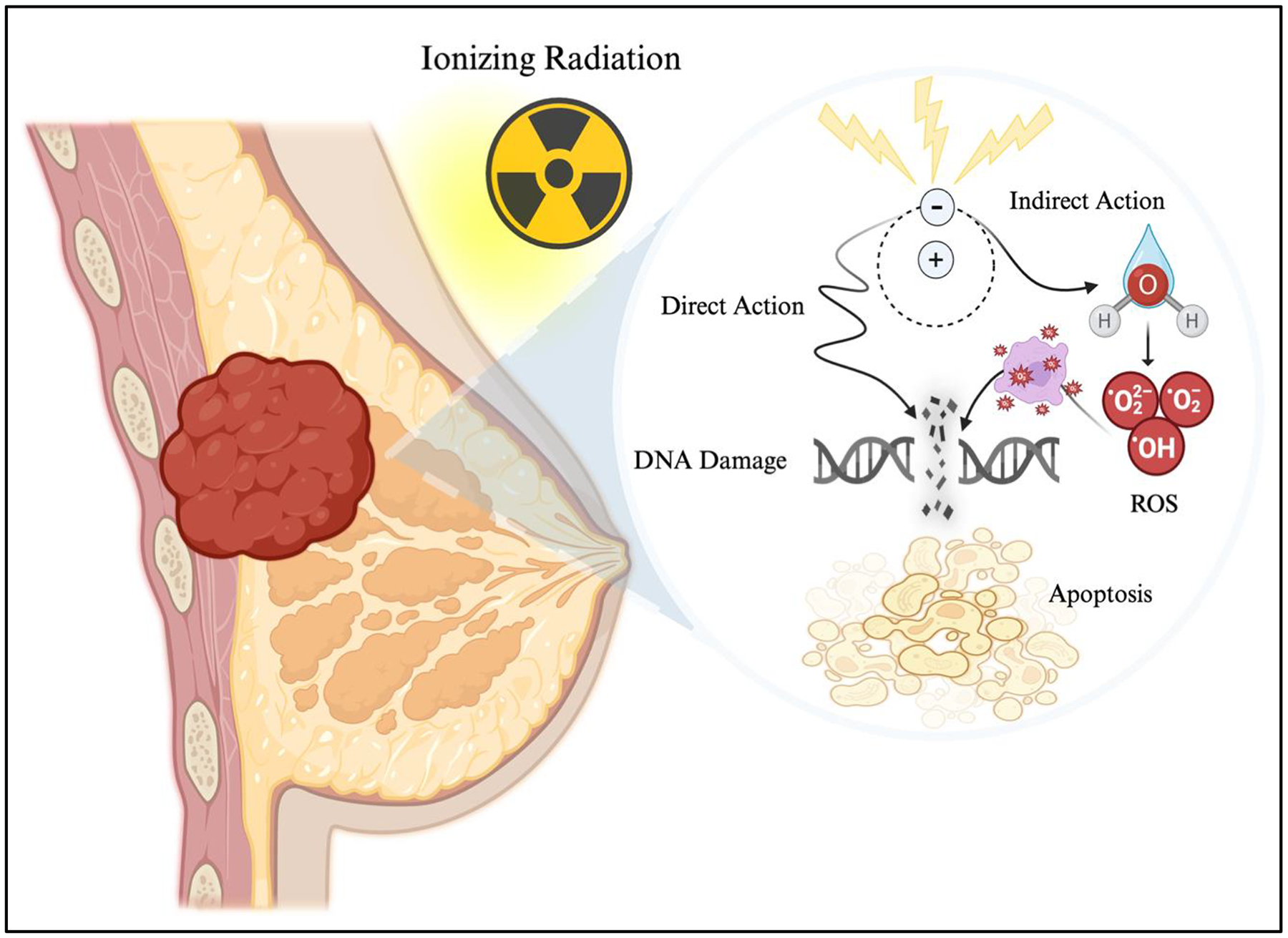
Radiotherapy-induced cytotoxicity. Ionizing radiation works through both direct and indirect mechanisms. Directly, radiation inflicts double-stranded DNA breaks, leading to cellular apoptosis. Indirectly, ionizing radiation generates reactive oxygen species (ROS) through water molecule ionization, which further damages cellular components, including DNA. Both pathways contribute to the cytotoxic effects of radiotherapy on cancer cells but also harm surrounding healthy tissues, increasing the risk of secondary malignancies, fibrosis, and other radiation-related complications. Created with BioRender.com.

The effectiveness of radiotherapy in treating breast cancer stems from its ability to induce DNA damage in rapidly proliferating cancer cells, primarily through the infliction of double-stranded breaks (DSBs) in DNA [16, 29]. This lethal mechanism is central to the cytotoxic effects of radiotherapy, leading to cell death if not sufficiently repaired [29,30]. Moreover, the cytotoxicity of radiotherapy is driven by direct and indirect pathways, as illustrated in Figure 1. Both can inadvertently damage the DNA of normal cells in the surrounding tissue and cause significant side effects, such as chromosomal aberrations and an increased risk for cardiac and pulmonary toxicity, and secondary malignancies [31,32].

In the direct mechanism, ionizing radiation interacts with the DNA molecules directly, breaking the phosphate backbone and creating DSBs [31]. For cancer cells, this kind of damage is particularly difficult to repair, with defects present in DNA damage response (DDR) pathways [33]. This commonly leads to cell death either through apoptosis or mitotic catastrophe, a process whereby cells fail to undergo typical chromosomal segregation and division [34,35]. While normal cells in the irradiated area can also suffer from DSBs, they can more readily attempt to repair the damage through various mechanisms, such as single-strand alignment, non-homologous end joining, and conservative homologous recombination [36]. Nevertheless, they remain highly vulnerable, as this damage can result in cell death and, in some cases, new malignancies due to improper repair [37].

The indirect mechanism involves the ionization of water molecules within the cell, producing reactive oxygen species (ROS), including hydroxyl radical, superoxide, and hydrogen peroxide [35,38]. These ROS overwhelm the cell’s natural antioxidant defenses and induce oxidative stress by attacking cellular components like lipids, proteins, and nucleic acids [39,40]. In cancer cells, such oxidative damage contributes to the formation of single-strand breaks (SSBs) and DSBs, driving cell death. In normal cells, however, the oxidative stress can also lead to chronic inflammation and tissue alterations, perpetuating a cycle of damage that extends beyond the initial exposure to radiation and potentially compromising reconstructive success [41].

The effects of post-mastectomy radiotherapy can manifest both acutely and in the long-term, with profound implications for reconstructive outcomes. Acute effects typically include fatigue, sore throat, and radiation dermatitis [16,42]. Radiation dermatitis is experienced by as many as 95% of patients within days to weeks of receiving radiotherapy, characterized by symptoms such as skin erythema, dryness, rash, hyperpigmentation, and moist desquamation arising from the immediate inflammatory response to cellular damage [43]. While these effects typically resolve within weeks, they set the stage for more severe, long-term complications that may not emerge until months, or even years after radiotherapy and reconstruction, including fibrosis, fat necrosis, tissue atrophy, and lymphedema [44–46].

**Figure 2:**
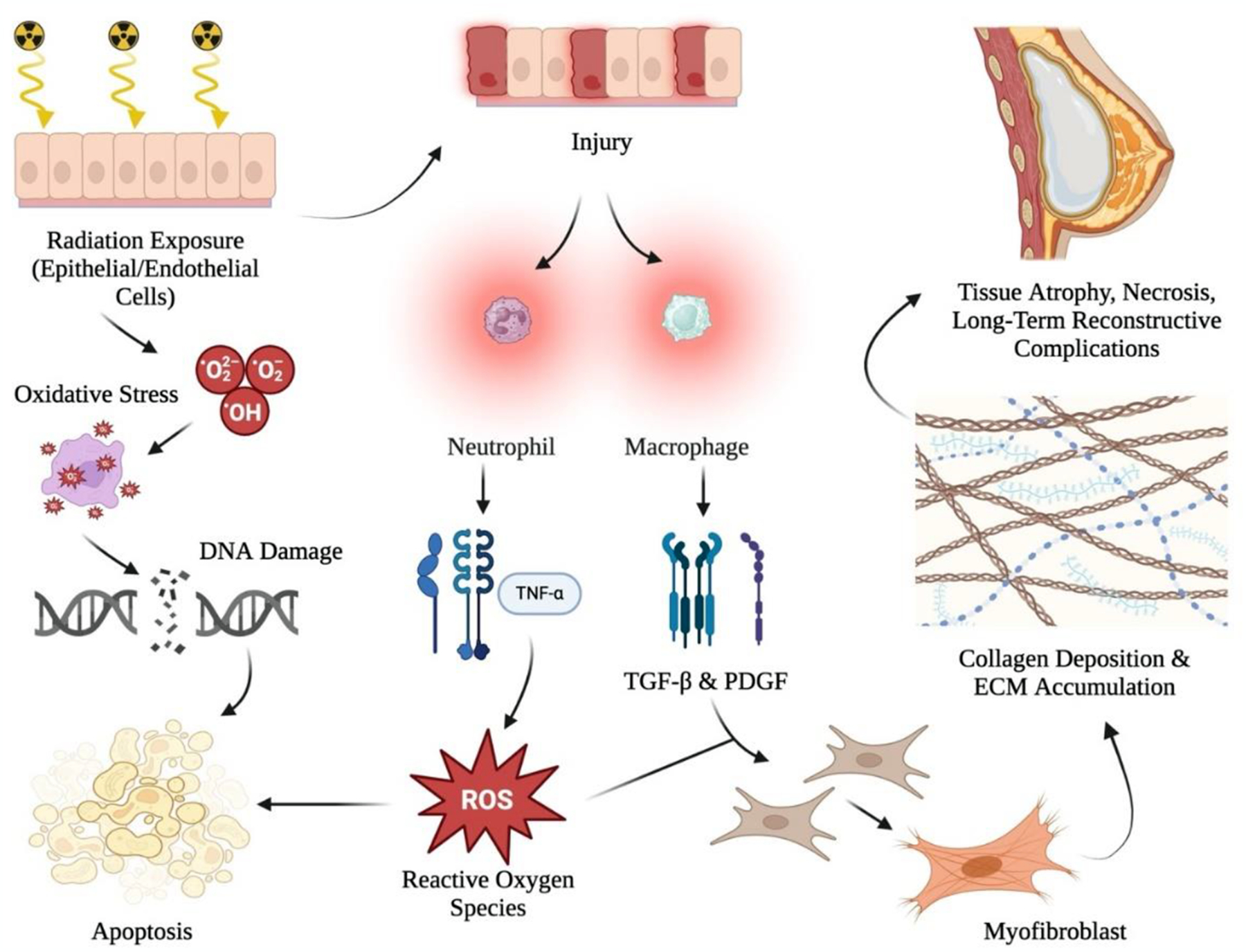
A comprehensive overview of the pathogenesis of radiation-induced tissue injury and fibrosis. Radiation exposure to epithelial and endothelial cells triggers oxidative stress, resulting in DNA damage and the initiation of apoptosis. This injury releases chemokines that recruit neutrophils and macrophages to the affected tissue, which subsequently release pro-inflammatory cytokines such as TNF-α, as well as growth factors like TGF-β and PDGF. These factors contribute to the excessive formation of reactive oxygen species (ROS), which further exacerbates tissue damage and cell death. Over time, fibroblasts differentiate into myofibroblasts, leading to collagen deposition and accumulation in the extracellular matrix (ECM). These changes, characterized by fibrosis, result in tissue stiffness, atrophy, necrosis, and long-term reconstructive complications such as capsular contracture, breast asymmetry, and compromised aesthetic outcomes. Created with BioRender.com.

Fibrosis, one of the most debilitating delayed effects, results from a complex cascade of cellular and molecular events that transform acute injury into chronic tissue scarring (Figure 2). The pathogenesis of radiation-induced fibrosis progresses through three stages. First, the inflammatory stage occurs when ionizing radiation triggers the release of pro-inflammatory cytokines like TNF-α, IL-1, and IL-6, which further promote ROS generation [47–49]. In the fibrotic stage, fibroblasts are activated and recruited by cytokines such as PDGF and TGF-β, leading to their differentiation into myofibroblasts and the excessive production of extracellular matrix (ECM) components like collagen. Finally, the fibro-atrophic stage is marked by tissue thickening, microvascular damage, and ischemia, contributing to tissue necrosis and atrophy. Another significant long-term effect of radiotherapy, fat necrosis, involves the death of adipose tissue [50,51]. As will be discussed, this is particularly relevant for autologous tissue reconstruction, where the tissue may have a compromised blood supply [52]. Moreover, fat necrosis not only affects the aesthetic outcome of breast reconstruction, but can also necessitate additional surgical interventions to remove necrotic tissue [50].

Given the severity of radiation-induced fibrosis and fat necrosis, therapeutic strategies that target the underlying pathologies of radiation-induced complications are critical for improving patient outcomes. Effective strategies could focus on inhibiting TGF-β signaling, neutralizing ROS, and restoring overall vascular integrity. In this context, radioprotective agents emerge as a promising option. However, before delving into an overview of prospective agents, it is important to first examine the impact of radiotherapy on different reconstructive techniques. This understanding will serve as a foundation for then discussing how radioprotective agents can be integrated into PMRT protocols to optimize surgical results.

## Impact on Reconstructive Techniques

Although post-mastectomy radiotherapy (PMRT) is integral in reducing locoregional recurrence in breast cancer patients, it can significantly affect functional and aesthetic outcomes after reconstruction. This impact varies, however, based on the type of reconstruction (implant-based or autologous), as well as the timing of reconstruction (immediate or delayed).

Implant-based reconstruction is often favored for its shorter recovery period and the absence of donor-site morbidity that can result from an autologous tissue transfer. Nevertheless, the interaction between PMRT and implant-based reconstruction can introduce various complications. Capsular contracture, a condition where a fibrous capsule forms and tightens around the implant, is one of such complications commonly associated with PMRT. This tightening can lead to pain, distortion of breast shape, and additional corrective surgeries [53]. Furthermore, current literature indicates that patients who undergo both PMRT and implant-based reconstruction are at a markedly higher risk of developing capsular contracture, experiencing reconstructive failure, and being less satisfied with the cosmetic outcome, regardless of the timing of the procedure relative to radiotherapy [54–57]. Additionally, PMRT can lead to numerous other complications that impact the outcomes of implant-based reconstruction. Radiotherapy induces acute toxicity, which increases the risk of wound complications such as infection, dehiscence, seroma, and delayed healing [24,58]. These skin changes are particularly problematic in prepectoral implant-based breast reconstruction, where the implant is placed directly beneath the skin without vascularized muscle coverage. Moreover, as described previously and illustrated in Figure 2, PMRT causes a chronic inflammatory response in irradiated tissue, leading to the deposition of excess collagen and subsequent fibrosis. This process can stiffen the skin and subcutaneous tissues surrounding the implant, reducing their elasticity and pliability. The result is a firmer, less natural breast contour, increased asymmetry, and a higher likelihood of implant malposition [59,60]. These fibrotic changes detract from the overall aesthetic outcome and patient satisfaction. The functional implications could also involve restricted movement and chronic discomfort, which can significantly impact daily activities and well-being. Interestingly, the addition of fat grafting to traditional implant-based reconstruction has shown potential in mitigating some of these complications, offering a way to improve outcomes [60]. Another common strategy to reduce complications is a two-stage approach involving tissue expanders followed by permanent implants. Tissue expanders are initially placed beneath the skin to gradually stretch the tissue, and once PMRT is complete, they are replaced with permanent implants [61]. This strategy allows the tissue to recover from radiation before final reconstruction, potentially lowering the risk of capsular contracture and improving overall aesthetic outcomes. However, these outcomes are influenced by the specific timing of expander-implant exchange [62].

Autologous tissue reconstruction, on the other hand, involves using the patient’s own tissue, often retrieved from the back, abdomen, buttock, or thigh, to reconstruct the breast. This technique decreases the rate of complications, such as capsular contracture and mastectomy skin flap necrosis, relative to implant-based [55]. Consequently, autologous reconstruction remains the preferred choice for patients with previously irradiated chests, or those planning to undergo radiotherapy [63–65]. Nonetheless, this approach still carries its own set of flaws. Fat necrosis, for instance, is a common complication in the context of autologous reconstruction [66,67]. This occurs when irradiated adipose tissue undergoes ischemia and subsequent cell death, forming firm, painful masses that can compromise the aesthetic and functional outcomes of the reconstruction. Radiation can also impair the microvascular networks within the transferred tissue, increasing the risk of flap loss, or vascular thrombosis [68,69]. This is particularly concerning when autologous reconstruction is performed immediately after mastectomy, as the newly transferred tissue is highly susceptible to the damaging effects of radiation. Studies have consistently shown that autologous reconstructions performed after PMRT, specifically within an interval of about 12 months after radiotherapy, tend to have better outcomes, as the tissue is not exposed to radiation while healing from the reconstructive procedure [70]. However, even in delayed reconstructions, the irradiated chest wall tissue can present challenges, including intraoperative vascular complications and impaired wound healing [69].

The timing of reconstruction, whether immediate or delayed, plays a critical role in determining the success of reconstruction. Numerous studies note the immense psychosocial benefit that immediate reconstruction can offer to patients [71–74]. Indeed, the immediate reconstruction, performed concurrently with mastectomy, exposes the newly reconstructed tissue to the effects of radiation, which can exacerbate complications such as capsular contracture in implants and fat necrosis. Delayed reconstruction, performed after the completion of radiotherapy, allows the tissues to recover from radiation-induced damage before undergoing the stress of reconstruction. This approach may significantly reduce the risk of complications [75]. Nevertheless, irradiated tissue can still present challenges such as altered vascularity, increased fibrosis, and reduced elasticity, making the surgical procedure more complex.

Ultimately, the choice of reconstruction technique and timing must be carefully accounted for, considering the patient’s oncologic status, the likelihood of requiring PMRT, and the potential impact on functional and aesthetic outcomes. A multidisciplinary approach involving oncologists, reconstructive surgeons, and radiation therapists is essential to optimize patient care and outcomes. As will be discussed in the next portion of this review, if the deleterious effects of radiotherapy can be minimized or even eliminated altogether, the potential for enhancing reconstructive outcomes and quality of life for breast cancer survivors becomes exponentially greater.

## Radioprotective Agents: An Overview

The adverse effects of ionizing radiation can be mitigated by several pharmacological agents. These agents are in general classified according to the timing of administration and differential biological responses. Three major classes of the modifiers of ionizing radiation include radiosensitizers, radiomitigators, and radioprotectors. Radiosensitizing agents are usually administered during radiotherapy to maximize its killing effect on tumor cells by accelerating DNA damage. Radiomitigators are administered in parallel to radiation exposure or post-radiation to attenuate the adverse effects. Radioprotective agents are garnering attention for their potential to mitigate the adverse effects of post-mastectomy radiotherapy (PMRT), whilst sustaining and enhancing oncologic efficacy.

**Figure 3:**
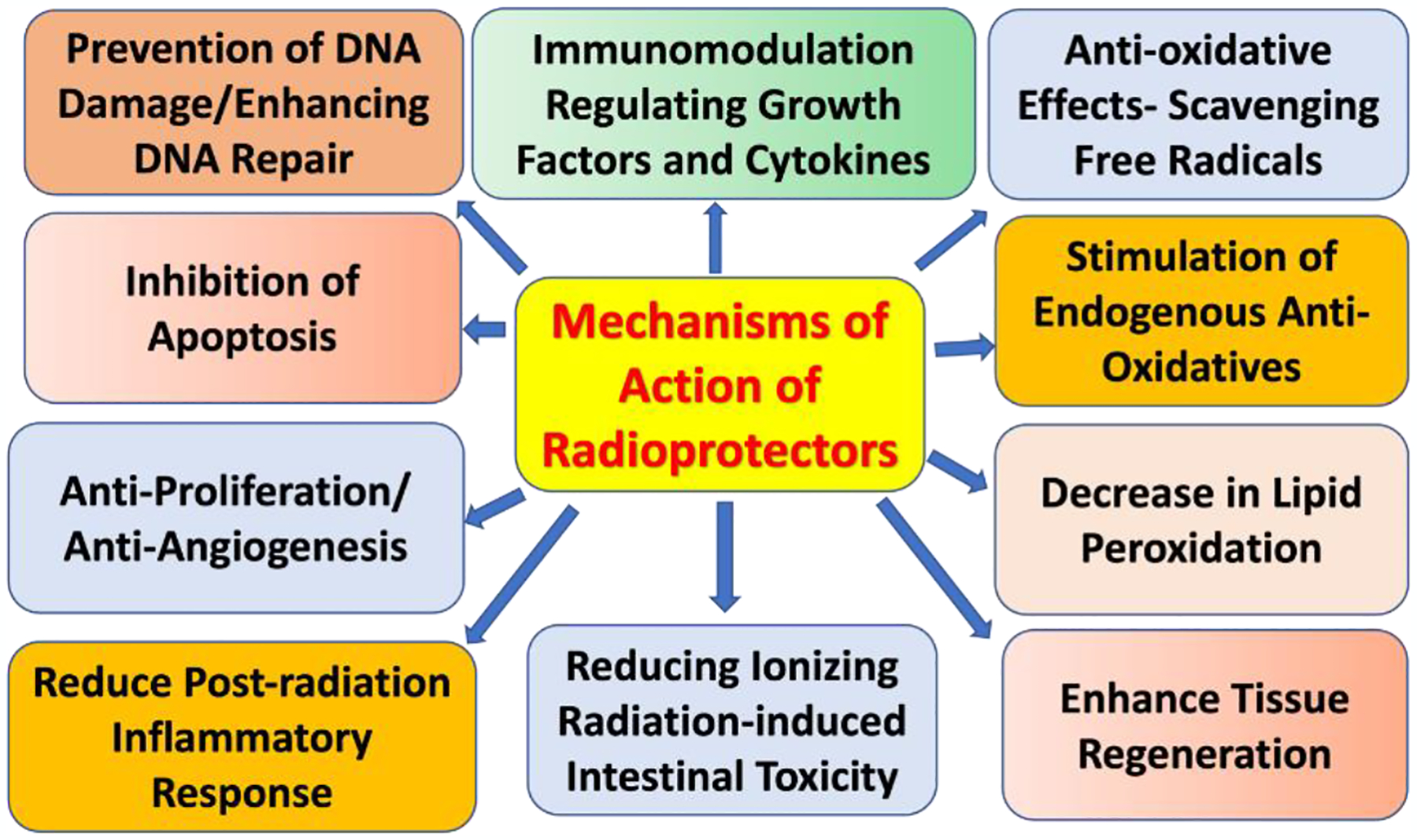
Schematic diagram showing the potential mechanisms of action of radioprotectors to mitigate adverse effects of ionizing radiation and enhance efficacy of radiotherapy.

In the following section, we will focus on radioprotective agents since these agents are crucial in the context of breast reconstruction, as radiation-induced complications can impair both functional and aesthetic outcomes. Radioprotective agents induce protective and beneficial effects via several mechanisms, including free radical scavengers, antioxidants, immunomodulation, anti-inflammatory, anti-fibrotic, anti-proliferative, anti-angiogenesis, prevention of DNA damage and enhancing DNA repair, decrease lipid peroxidation (Figure 3). Thus, radioprotective agents offer promising strategies to protect tissues from the adverse effects of ionizing radiation during radiotherapy (Figure 3).

Indeed, many radioprotectors have been reported in the literature. These include natural products such as flavonoid and non-flavonoid polyphenols, polysaccharides, synthetic molecules, DNA-binding agents, hormones, cytokines, nitroxides such as tempol, immune modulators, autophagy inhibitors, vitamins, metformin, inhibitors of poly-(adenosine diphosphate-ribose)-polymerase (PARP), and others. The goal is to use an effective radioprotector with minimal adverse effects of its own or the ionizing radiation to normal tissue and enhance efficacy of the radiotherapy in the management of breast cancer. Accordingly, in the following section we discussed the role of selected radioprotective agents, such as amifostine, N-acetylcysteine (NAC), tempol, manganese superoxide dismutase (MnSOD) plasmid liposomes, vitamin E, and beta-carotene, with a focus on their logistical applications in breast reconstruction.

## Amifostine

**Figure 4:**
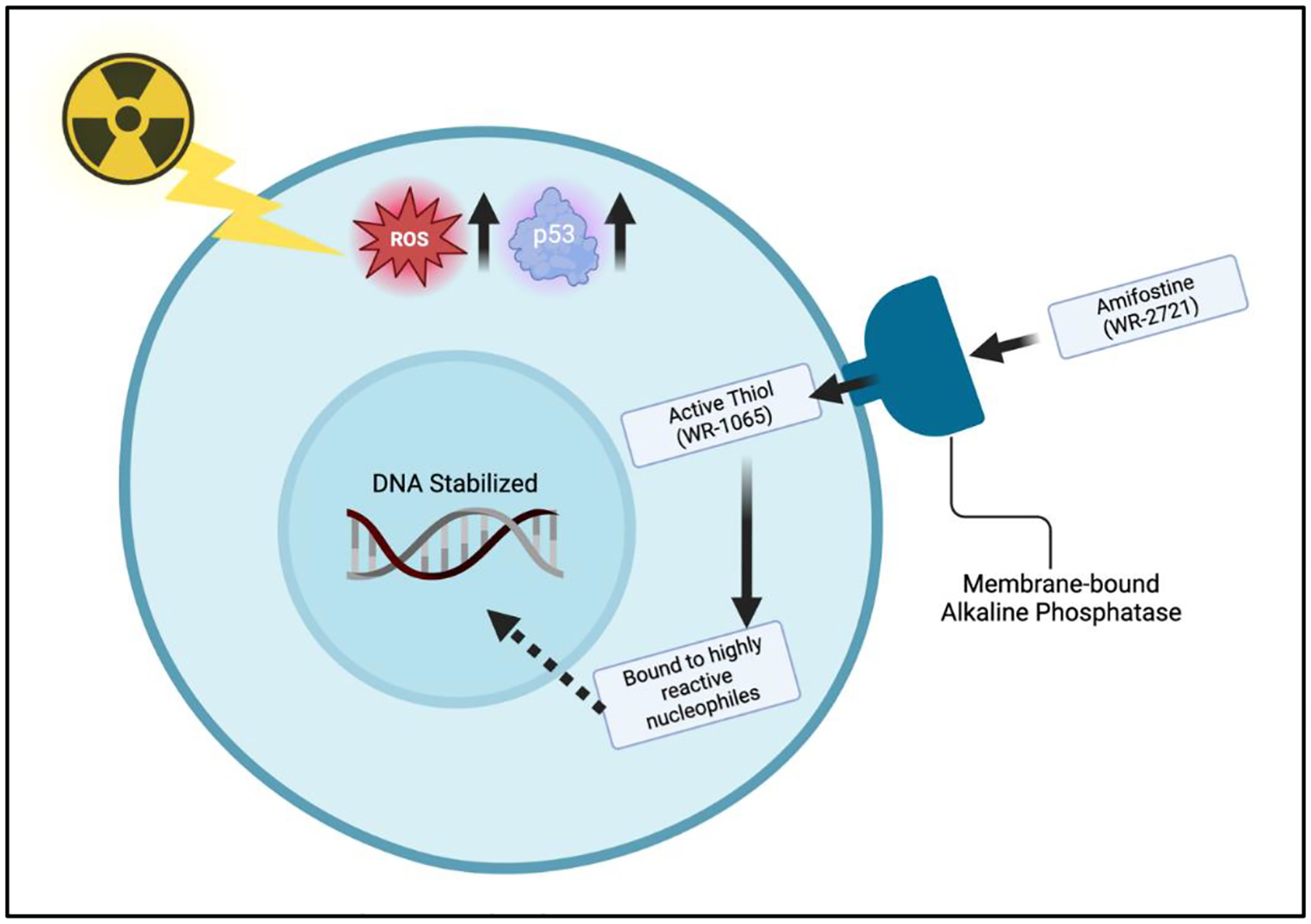
The mechanism of action of amifostine, a radioprotective agent. After administration, amifostine is converted into its active thiol metabolite through dephosphorylation by membrane-bound alkaline phosphatase, which is predominantly found in healthy tissues. This active form binds to highly reactive nucleophiles, stabilizing DNA and mitigating radiation-induced damage. By scavenging reactive oxygen species (ROS) and increasing p53 levels, amifostine protects normal tissues from apoptosis and other radiation-induced effects, while its uptake in tumor cells is limited. Created with BioRender.com.

Amifostine is among the most extensively researched radioprotective agents, primarily functioning as a prophylactic free radical scavenger which neutralizes the reactive oxygen species (ROS) generated during radiotherapy. Its selective accumulation in normal tissues is facilitated by its uptake through membrane-bound alkaline phosphatase, predominantly in healthy cells (Figure 4) [76,77]. Due to a deficiency in such enzymes, the uptake of amifostine by tumor cells is extremely limited. This selectivity is critical, allowing amifostine to significantly reduce radiation-induced complications, particularly those affecting the skin and soft tissues, without compromising oncologic outcomes [78]. In breast reconstruction, where post-mastectomy radiotherapy (PMRT) often results in complications such as fibrosis, capsular contracture, and impaired wound healing, amifostine presents a promising adjunct to mitigate these effects. Although its oncologic safety in breast cancer has been established in vitro, further clinical trials and investigation in vivo are warranted to solidify its role in optimizing outcomes in breast reconstruction after radiotherapy [28].

## N-Acetylcysteine

N-Acetylcysteine (NAC) is a free radical scavenger known for its ability to replenish intracellular levels of glutathione, one of the most powerful endogenous antioxidants, and enhance the neutralization of oxidative stress in the cell [79]. NAC effectively decreases ROS production and inhibits ROS-mediated signaling, which contribute to cancerous cell survival and metastasis in breast cancer [26,27]. Additionally, several studies involving in-vitro assays and animal models have shown that NAC bears the potential to protect against a number of radiation-induced injuries, including radiation dermatitis, reduced skin elasticity, and impaired wound healing [80–83]. In the context of breast reconstruction, NAC could be particularly valuable in autologous tissue reconstructions where radiation often impairs tissue perfusion, increasing the risk of fat necrosis. By improving tissue oxygenation and reducing fibrosis, NAC can help maintain the pliability and appearance of the reconstructed breast, enhancing both functional and aesthetic outcomes. Furthermore, NAC may aid in preventing capsular contracture in implant-based reconstructions by reducing inflammatory cytokine production and collagen deposition [84].

## Tempol

Tempol is a free radical nitroxide that acts as a potent ROS scavenger and antioxidant, neutralizing superoxide radicals and protecting tissues from oxidative stress [85]. Tempol has been studied extensively, demonstrating efficacy in reducing radiation-induced skin toxicity and fibrosis [86,87]. Such complications are common in patients undergoing PMRT and can significantly compromise breast reconstruction outcomes. In implant-based approaches, where the implant is placed pre-pectorally without muscle coverage, the skin and subcutaneous tissues are more vulnerable to radiation damage. The ability of tempol to reduce oxidative damage may help preserve skin elasticity and vascular integrity, reducing complications like skin necrosis, wound dehiscence, and implant exposure. Given these benefits, tempol could improve both the functional and aesthetic results of breast reconstruction by minimizing radiation-induced tissue damage.

## Manganese Superoxide Dismutase Plasmid Liposomes

Manganese superoxide dismutase (MnSOD) plasmid liposomes represent a gene therapy-based approach to radioprotection. MnSOD is a mitochondrial enzyme that converts superoxide radicals into less harmful molecules like hydrogen peroxide and oxygen, thus protecting tissues from oxidative stress [88]. By enhancing the natural endogenous antioxidant defense, MnSOD has been shown to reduce radiation-induced fibrosis and skin damage, protecting microvascular integrity in normal tissues [89–91]. In breast reconstruction, MnSOD plasmid liposomes may be especially beneficial in autologous tissue reconstructions, where flap viability is closely tied to the integrity of the microvascular networks. As mentioned earlier, radiation-induced microvascular damage can result in ischemia, fat necrosis, and flap failure. The use of MnSOD could help prevent these complications, improving flap survival and reducing the need for revision surgeries. By protecting the vascular system, MnSOD also holds promise in enhancing the cosmetic outcomes of autologous reconstructions.

## Glutamine

Glutamine is an abundant amino acid that plays a critical role in preventing free radical damage and maintaining tissue integrity. In radiotherapy, glutamine supplementation has been studied for its potential to reduce radiation-induced mucositis and enhance recovery in irradiated tissues [92]. With breast reconstruction, glutamine’s ability to support tissue repair and reduce inflammation could help mitigate complications such as necrosis and poor wound healing, particularly in irradiated tissue flaps [93,94]. Furthermore, this antioxidant could contribute to faster healing and reduced fibrosis in implant-based reconstruction, helping to maintain the aesthetic contour of the breast. In autologous reconstructions, it may improve flap survival by promoting microvascular recovery and reducing ischemic injury.

## Pentoxifylline

Pentoxifylline (PTX) is a vasodilator with anti-inflammatory properties that has been used to reverse established radiation-induced fibrosis when combined with vitamin E [95–97]. By enhancing blood flow and reducing collagen deposition and inflammation, PTX could potentially improve tissue elasticity and the incidence of capsular contracture in implant-based reconstructions [98,99]. In autologous reconstructions, PTX may help prevent fat necrosis and improve flap viability by promoting microvascular integrity and reducing ischemic injury.

## Vitamin E

Vitamin E is a powerful antioxidant that protects cell membranes from radiation-induced oxidative damage [100]. Its radioprotective effects have been studied in the context of skin toxicity, and it has been shown to reduce the severity of radiation dermatitis and fibrosis [13,101]. In breast reconstruction, vitamin E may help preserve the softness and elasticity of irradiated tissues, reducing the risk of capsular contracture and improving overall aesthetic outcomes. Moreover, when combined with PTX, vitamin E has been shown to reverse established fibrosis, making it a valuable agent in managing radiation-induced complications in breast reconstruction [95].

## Integration of Radioprotective Agents into Clinical Practice

The integration of radioprotective agents into post-mastectomy radiotherapy (PMRT) protocols presents an opportunity to critically advance and optimize reconstructive outcomes, with the potential to simultaneously reduce complications and preserve oncologic efficacy. Although there are many others that have been investigated, radioprotective agents such as amifostine, N-acetylcysteine, tempol, MnSOD plasmid liposomes, vitamin E, and glutamine offer promising strategies to mitigate the damage caused by PMRT, due to their respective mechanisms of action. However, successfully integrating these agents into clinical practice involves addressing key factors such as optimal timing, dosage, patient selection, and potential combination therapies.

## Optimal Timing and Dosage

The efficacy of radioprotective agents is highly dependent on their timing and dosage. Agents like amifostine exhibit peak effectiveness when administered prophylactically before radiation exposure, allowing for selective accumulation in normal tissues and the neutralization of reactive oxygen species (ROS) before significant damage occurs [28]. In implant-based reconstructions specifically, the strategic use of radioprotective agents, either before or after shortly after radiation, can help reduce associated complications [102]. Evaluating the dose reduction factor, which quantifies the effectiveness of a given radioprotector, ranges from 40 to 200 rads/min [103]. Nevertheless, the optimal dosage for each agent remains to be fully defined, necessitating careful consideration of patient-specific factors such as radiation dose, tissue characteristics, and underlying comorbidities, to inform clinical decisions regarding the most appropriate dosage and timing [104].

## Patient Selection Criteria

Patient selection is critical to the effective use of radioprotective agents, as those at the highest risk for radiation-induced complications like capsular contracture, fat necrosis, or impaired wound healing, are prime candidates to receive such therapies. Individuals with poor skin elasticity, pre-existing fibrosis, or those undergoing pre-pectoral implant-based reconstruction, which entails increased tissue vulnerability due to the lack of vascularized muscle coverage, may particularly benefit from agents like N-acetylcysteine and tempol, as they work to mitigate oxidative damage and support tissue integrity. Similarly, in autologous reconstructions, where ischemic injury poses a significant risk for flap failure, agents such as MnSOD plasmid liposomes and glutamine hold promise for enhancing microvascular function and promoting flap survival. By tailoring radioprotective therapies to the specific risks and reconstructive techniques of each patient, clinicians may enhance both functional and aesthetic outcomes, underscoring the importance of individualized treatment planning in post-mastectomy radiotherapy protocols.

## Combination Therapies

Combining radioprotective agents with one another, or even various other therapeutic modalities, holds considerable promise in enhancing reconstructive outcomes. For instance, the combination of antioxidants, such as vitamin E and pentoxifylline (PTX), has demonstrated synergistic benefits like the reversal of radiation-induced fibrosis, offering potential solutions for managing capsular contracture and improving tissue elasticity [95]. While these combination therapies appear beneficial, further clinical trials are required to fully understand their safety, efficacy, and appropriate clinical applications in the context of breast reconstruction.

## Cost-effectiveness and Ethical Considerations

While radioprotective agents present clear clinical benefits, cost and accessibility must be accounted for before integration into standard practice. Agents, particularly synthetic chemical compounds, are associated with high production and processing costs [105]. This raises concerns about equitable access in resource-limited settings. Moreover, ethical considerations regarding the allocation of these therapies and ensuring that patients with limited financial means still receive high-quality care, are crucial. Policymakers and healthcare providers must thoroughly weigh the long-term benefits of radioprotective agents, such as reduced rates of revision surgery and improved quality of life, against their upfront costs.

## Challenges and Future Directions

Despite the potential of radioprotective agents to transform reconstructive outcomes following PMRT, their clinical integration faces several challenges. Foremost among these is the lack of robust, large-scale randomized controlled trials to establish standardized protocols for the timing, dosage, and combination of these agents. The current literature, while promising, largely consists of preclinical studies and small clinical trials, many of which focus on singular agents. To ensure these therapies become a mainstay in breast reconstruction, future research must focus on large-scale trials that include diverse patient populations to determine long-term safety and efficacy. Another key challenge is the identification of novel radioprotective agents. While some agents have demonstrated effectiveness in reducing radiation-induced damage, there remains a need for newer agents with improved selectivity and fewer side effects. The ongoing development of agents targeting specific molecular pathways, such as inhibitors of TGF-β signaling, could offer more targeted radioprotection, minimizing damage to normal tissues and preserving oncologic efficacy. Investigating the application of radioprotective agents with emerging technologies like proton therapy or advanced imaging-guided radiotherapy presents another area for future research. Furthermore, the successful integration of radioprotective agents into PMRT protocols necessitates a collaborative, multidisciplinary approach involving oncologists, reconstructive surgeons, and rehabilitation specialists. Close coordination among these professionals is crucial to developing individualized treatment plans that incorporate radioprotective agents effectively, while also ensuring that oncologic outcomes are maintained. Multidisciplinary teams can optimize the timing of radiotherapy and surgery, select appropriate radioprotective agents, and adjust dosages based on patient-specific factors. This approach fosters holistic patient care, which is essential for improving both the functional and aesthetic outcomes of breast reconstruction [7]. Finally, the development of personalized treatment approaches is crucial for maximizing the benefits of prospective agents. Given the variability in patient responses to both radiotherapy and radioprotective modalities, personalized medicine offers an opportunity to tailor these therapies to individual needs, considering genetic, environmental, and clinical factors. Future research could even focus on the identification of biomarkers that predict patient response to radioprotective agents, further enabling the development of customized treatment plans that optimize both reconstructive and oncologic outcomes.

To summarize, while the integration of radioprotective agents into PMRT protocols offers significant potential to improve reconstructive outcomes, considerable challenges remain. Addressing these challenges through robust clinical research, novel agent development, multidisciplinary collaboration, and personalized treatment strategies will be crucial to fully realizing the promise of radioprotective agents in breast reconstruction. As the field continues to evolve, the combined efforts of clinicians and scientists will be integral to ensuring that these advances translate into improved outcomes and quality of life for breast cancer survivors.

## Figures and Tables

**Table 1:** Prospective Radioprotective agents and the underlying mechanisms of action.

Radioprotective Agent	Mechanism of Action	Reference
Amifostine	Free radical scavenger and DNA repair agent, detoxifying harmful ROS generated by radiation	[76]
N-Acetylcysteine (NAC)	Increases intracellular levels of glutathione, neutralizing ROS	[78]
Tempol	Free radical scavenger and neutralizing agent for superoxide	[84]
Manganese Superoxide Dismutase (MnSOD) Plasmid Liposomes	Converts superoxide radicals to less harmful molecules, reducing oxidative stress	[87]
Glutamine	Detoxifies and prevents free radical damage as an antioxidant	[91]
Pentoxifylline (PTX)	Anti-inflammatory, vasodilator, and immunomodulator	[96]
Vitamin E	Stabilizes cell membrane and scavenges free radicals, as an antioxidant	[99]

## Key points

- Indeed, post-mastectomy radiotherapy reduces the risk of reoccurrence of breast cancer but may induce adverse effects.
- Radioprotective agents can mitigate the adverse effects of ionizing radiation and improve the outcomes in breast cancer patients, reduce relapse rates, and decrease morbidity and mortality.
- Integration of radioprotective agents into post-mastectomy radiotherapy protocols offers significant potential to improve reconstructive outcomes.
- The characteristics of an ideal radioprotective agent include protective effect before or after radiotherapy with high activity, high selectivity, and low toxic side effects, prevention and/or repair of tissue damage following radiotherapy, and rapid onset with long-half life.
- Radioprotective agents induce protective and beneficial effects via several mechanisms, including free radical scavengers, antioxidants, immunomodulation, anti-inflammatory, anti-fibrotic, anti-proliferative, anti-angiogenesis, prevention of DNA damage and enhancing DNA repair, decrease lipid peroxidation.
- There is a need to identify biomarkers that can predict response to radioprotective agents to optimize both reconstructive and oncologic outcomes and thus enhance the development of customized treatment plans.
- There is a need to develop effective radioprotective agent that can be given orally.
- Well-designed and placebo-controlled clinical trials are warranted to fully understand the safety, efficacy, and clinical application of radioprotective agents in breast reconstruction.

## References

[1] Łukasiewicz S, Czeczelewski M, Forma A, Breast Cancer-Epidemiology, Risk Factors, Classification, Prognostic Markers, and Current Treatment Strategies - An Updated Review. Cancers 13 (2021): 4287. 34503097 10.3390/cancers13174287 PMC8428369

[2] Bray F, Laversanne M, Sung H, Global cancer statistics 2022: GLOBOCAN estimates of incidence and mortality worldwide for 36 cancers in 185 countries. CA Cancer J Clin 74 (2024): 229–263. 38572751 10.3322/caac.21834

[3] Francies FZ, Hull R, Khanyile R, Breast cancer in low-middle income countries: abnormality in splicing and lack of targeted treatment options. Am J Cancer Res 10 (2020): 1568–1591. 32509398 PMC7269781

[4] Wilkinson L, Gathani T. Understanding breast cancer as a global health concern. Br J Radiol 95 (2022): 20211033. 34905391 10.1259/bjr.20211033 PMC8822551

[5] Hoxha I, Islami DA, Uwizeye G, Forty-Five Years of Research and Progress in Breast Cancer: Progress for Some, Disparities for Most. JCO Glob Oncol 8 (2022): e2100424. 35377728 10.1200/GO.21.00424 PMC9005254

[6] Saini KS, Taylor C, Ramirez AJ, Role of the multidisciplinary team in breast cancer management: results from a large international survey involving 39 countries. Ann Oncol 23 (2012): 853–859. 21821551 10.1093/annonc/mdr352

[7] Leclerc AF, Jerusalem G, Devos M, Multidisciplinary management of breast cancer. Arch Public Health 74 (2016): 50. 27980734 10.1186/s13690-016-0163-7 PMC5137213

[8] Shao J, Rodrigues M, Corter AL, Multidisciplinary Care of Breast Cancer Patients: A Scoping Review of Multidisciplinary Styles, Processes, and Outcomes. Curr Oncol 26 (2019): 385–397. 10.3747/co.26.4713 PMC6588064 31285683

[9] Cheifetz R, McKevitt E. Advances in the Surgical Treatment of Breast Cancer. Curr Oncol 30 (2023): 9584–9586. 37999113 10.3390/curroncol30110693 PMC10670006

[10] Riis M. Modern surgical treatment of breast cancer. Ann Med Surg 56 (2020): 95–107. 10.1016/j.amsu.2020.06.016 PMC7327379 32637082

[11] Wang J, Wu SG. Breast Cancer: An Overview of Current Therapeutic Strategies, Challenge, and Perspectives. Breast Cancer Targets Ther 15 (2023): 721–730. 10.2147/BCTT.S432526 PMC10596062 37881514

[12] Katsura C, Ogunmwonyi I, Kankam HK, Breast cancer: presentation, investigation and management. Br J Hosp Med 83 (2022): 1–7. 10.12968/hmed.2021.0459 35243878

[13] Kayali M, Abi JJ, Ramia P, Post-lumpectomy radiation therapy boost in breast cancer patients: evidence revisited. Ecancermedicalscience 15 (2021): 11. 10.3332/ecancer.2021.1194 PMC8043677 33889203

[14] Walsh SM, Lowery AJ, Prichard RS, Postmastectomy radiotherapy: Indications and implications. The Surgeon 12 (2014): 310–315. 25037652 10.1016/j.surge.2014.04.004

[15] Ebctcg (Early Breast Cancer Trialists’ Collaborative Group). Effect of radiotherapy after mastectomy and axillary surgery on 10-year recurrence and 20-year breast cancer mortality: meta-analysis of individual patient data for 8135 women in 22 randomised trials. The Lancet 383 (2014): 2127–2135. 10.1016/S0140-6736(14)60488-8 PMC5015598 24656685

[16] Arnautovic A, Olafsson S, Wong JS, Optimizing Breast Reconstruction through Integration of Plastic Surgery and Radiation Oncology. Plast Reconstr Surg - Glob Open 9 (2021): e3577. 33977003 10.1097/GOX.0000000000003577 PMC8104197

[17] Overgaard M, Nielsen HM, Tramm T, Postmastectomy radiotherapy in high-risk breast cancer patients given adjuvant systemic therapy. A 30-year long-term report from the Danish breast cancer cooperative group DBCG 82bc trial. Radiother Oncol 170 (2022): 4–13. 35288227 10.1016/j.radonc.2022.03.008

[18] Luo C, Zhong X, Fan Y, The effect of postmastectomy radiation therapy on high-risk patients with T1–2N0 breast cancer. The Breast 60 (2021): 1–5. 34455226 10.1016/j.breast.2021.08.006 PMC8399378

[19] Wei J, Jiang Y, Shao Z. The survival benefit of postmastectomy radiotherapy for breast cancer patients with T1–2N1 disease according to molecular subtype. The Breast 51 (2020): 40–49. 32200207 10.1016/j.breast.2020.03.003 PMC7375676

[20] Elawa S, Mirdell R, Stefanis A, Microcirculatory changes in the skin after postmastectomy radiotherapy in women with breast cancer. Sci Rep 14 (2024): 41–49. 38378732 10.1038/s41598-024-54650-4 PMC10879083

[21] Hubenak J, Zhang Q, Branch C, Mechanisms of Injury to Normal Tissue after Radiotherapy: A Review. J Plast Reconstr Surg 133 (2014): 49e–56e. 10.1097/01.prs.0000440818.23647.0b PMC3921068 24374687

[22] Jagsi R, Momoh AO, Qi J, Impact of Radiotherapy on Complications and Patient-Reported Outcomes After Breast Reconstruction. JNCI J Natl Cancer Inst 110 (2018): 157–165. 28954300 10.1093/jnci/djx148 PMC6059091

[23] Williams NR, Williams S, Kanapathy M, Radiation-induced fibrosis in breast cancer: A protocol for an observational cross-sectional pilot study for personalised risk estimation and objective assessment. Int J Surg Protoc 14 (2019): 9–13. 31851743 10.1016/j.isjp.2019.02.002 PMC6913559

[24] Jagsi R, Jiang J, Momoh AO, Complications After Mastectomy and Immediate Breast Reconstruction for Breast Cancer: A Claims-Based Analysis. Ann Surg 263 (2016): 219–227. 25876011 10.1097/SLA.0000000000001177 PMC4824182

[25] Hammond JB, Kosiorek HE, Cronin PA, Capsular contracture in the modern era: A multidisciplinary look at the incidence and risk factors after mastectomy and implant-based breast reconstruction. Am J Surg 221 (2021): 1005–1010. 32988607 10.1016/j.amjsurg.2020.09.020

[26] Allegra AG, Mannino F, Innao V, Radioprotective Agents and Enhancers Factors. Preventive and Therapeutic Strategies for Oxidative Induced Radiotherapy Damages in Hematological Malignancies. Antioxidants 9 (2020): 1116. 33198328 10.3390/antiox9111116 PMC7696711

[27] Smith TA, Kirkpatrick DR, Smith S, Radioprotective agents to prevent cellular damage due to ionizing radiation. J Transl Med 15 (2017): 232–249. 29121966 10.1186/s12967-017-1338-x PMC5680756

[28] Luby AO, Subramanian C, Buchman LK, Amifostine Prophylaxis in Irradiated Breast Reconstruction: A Study of Oncologic Safety In Vitro. Ann Plast Surg 85 (2020): 424–429. 31850964 10.1097/SAP.0000000000002110 PMC7295666

[29] Czajkowski D, Szmyd R, Gee HE. Impact of DNA damage response defects in cancer cells on response to immunotherapy and radiotherapy. J Med Imaging Radiat Oncol 66 (2022): 546–559. 35460184 10.1111/1754-9485.13413 PMC9321602

[30] Guo G, Zhang F, Gao R, DNA repair and synthetic lethality. Int J Oral Sci 3 (2011): 176–179. 22010575 10.4248/IJOS11064 PMC3469974

[31] Borrego-Soto G, Ortiz-López R, Rojas-Martínez A. Ionizing radiation-induced DNA injury and damage detection in patients with breast cancer. Genet Mol Biol 38 (2015): 420–432. 26692152 10.1590/S1415-475738420150019 PMC4763322

[32] Halyard M, Brown L, Mutter R. Benefits, risks, and safety of external beam radiation therapy for breast cancer. Int J Womens Health. Published online 4 (2015): 449. 10.2147/IJWH.S55552 PMC4418389 25977608

[33] Srinivas US. ROS and the DNA damage response in cancer. Redox Biol 11 (2019). 10.1016/j.redox.2018.101084 PMC6859528 30612957

[34] Chen H, Han Z, Luo Q, Radiotherapy modulates tumor cell fate decisions: a review. Radiat Oncol 17 (2022): 196. 36457125 10.1186/s13014-022-02171-7 PMC9714175

[35] Adjemian S, Oltean T, Martens S, Ionizing radiation results in a mixture of cellular outcomes including mitotic catastrophe, senescence, methuosis, and iron-dependent cell death. Cell Death Dis 11 (2020): 1003. 33230108 10.1038/s41419-020-03209-y PMC7684309

[36] Langerak P, Russell P. Regulatory networks integrating cell cycle control with DNA damage checkpoints and double-strand break repair. Philos Trans R Soc B Biol Sci 366 (2011): 3562–3571. 10.1098/rstb.2011.0070 PMC3203453 22084383

[37] De Gonzalez AB, Curtis RE, Kry SF, Proportion of second cancers attributable to radiotherapy treatment in adults: a cohort study in the US SEER cancer registries. Lancet Oncol 12 (2011): 353–360. 21454129 10.1016/S1470-2045(11)70061-4 PMC3086738

[38] Kyoung TJ, Park JW. The use of ebselen for radioprotection in cultured cells and mice. Free Radic Biol Med 46 (2009): 1177–1185. 19439217 10.1016/j.freeradbiomed.2009.01.023

[39] Schieber M, Chandel NS. ROS Function in Redox Signaling and Oxidative Stress. Curr Biol 24 (2014): R453–R462. 24845678 10.1016/j.cub.2014.03.034 PMC4055301

[40] Terasaki Y, Ohsawa I, Terasaki M, Hydrogen therapy attenuates irradiation-induced lung damage by reducing oxidative stress. Am J Physiol-Lung Cell Mol Physiol 301 (2011): L415–L426. 21764987 10.1152/ajplung.00008.2011

[41] Morry J, Ngamcherdtrakul W, Yantasee W. Oxidative stress in cancer and fibrosis: Opportunity for therapeutic intervention with antioxidant compounds, enzymes, and nanoparticles. Redox Biol 11 (2017): 240–253. 28012439 10.1016/j.redox.2016.12.011 PMC5198743

[42] Remick J, Amin N. Postmastectomy Breast Cancer Radiation Therapy. StatPearls Publishing (2023). 30085576

[43] Kim H, Kang D, Park W, Impact of Breast Reconstruction on Biophysical Parameters of Mammary Skin in Patients Receiving Postmastectomy Radiotherapy for Breast Cancer. J Breast Cancer 24 (2021): 206. 33913276 10.4048/jbc.2021.24.e23 PMC8090804

[44] Mirzabeigi M, Smartt J, Nelson J, An Assessment of the Risks and Benefits of Immediate Autologous Breast Reconstruction in Patients Undergoing Postmastectomy Radiation Therapy. Ann Plast Surg 71 (2013): 149–155. 23542828 10.1097/SAP.0b013e31824b3dcc

[45] Sigalove S. Prepectoral breast reconstruction and radiotherapy- a closer look. Gland Surg 8 (2019): 67–74. 30842930 10.21037/gs.2019.01.01 PMC6378256

[46] Ozaslan C, Kuru B. Lymphedema after treatment of breast cancer. Am J Surg 187 (2004): 69–72. 14706589 10.1016/j.amjsurg.2002.12.003

[47] Fijardo M, Kwan JYY, Bissey PA, The clinical manifestations and molecular pathogenesis of radiation fibrosis. eBioMedicine 103 (2024): 105089. 38579363 10.1016/j.ebiom.2024.105089 PMC11002813

[48] Straub JM, New J, Hamilton CD, Radiation-induced fibrosis: mechanisms and implications for therapy. J Cancer Res Clin Oncol 141 (2015): 1985–1994. 25910988 10.1007/s00432-015-1974-6 PMC4573901

[49] Wang B, Wei J, Meng L, Advances in pathogenic mechanisms and management of radiation-induced fibrosis. Biomed Pharmacother 121 (2020): 109560. 31739160 10.1016/j.biopha.2019.109560

[50] Janssen TJ, Wigley CH, Adegbie D, The treatment of symptomatic fat necrosis: A review and introduction of a new treatment algorithm. J Plast Reconstr Aesthet Surg 77 (2023): 87–93. 36563639 10.1016/j.bjps.2022.11.045

[51] Lövey K, Fodor J, Major T, Fat Necrosis After Partial-Breast Irradiation With Brachytherapy or Electron Irradiation Versus Standard Whole-Breast Radiotherapy-4-Year Results of a Randomized Trial. Int J Radiat Oncol 69 (2007): 724–731. 10.1016/j.ijrobp.2007.03.055 17524571

[52] Lee J, Park HY, Kim WW, Natural course of fat necrosis after breast reconstruction: a 10-year follow-up study. BMC Cancer 21 (2021): 166. 33593330 10.1186/s12885-021-07881-x PMC7885495

[53] Gorgy A, Barone N, Nepon H, Implant-based breast surgery and capsular formation: when, how and why?-a narrative review. Ann Transl Med 11 (2023): 385–385. 37970601 10.21037/atm-23-131 PMC10632565

[54] Ricci J, Epstein S, Momoh AO, A meta-analysis of implant-based breast reconstruction and timing of adjuvant radiation therapy. J Surg Res 218 (2017): 108–116. 28985836 10.1016/j.jss.2017.05.072

[55] Kronowitz S, Robb G. Radiation therapy and breast reconstruction: a critical review of the literature. J Plast Reconstr Surg 124 (2009): 395–408. 10.1097/PRS.0b013e3181aee987 19644254

[56] Cordeiro P, Albornoz C, McCormick B, The impact of postmastectomy radiotherapy on two-stage implant breast reconstruction: an analysis of long-term surgical outcomes, aesthetic results, and satisfaction over 13 years. J Plast Reconstr Surg 134 (2014): 588–595. 10.1097/PRS.0000000000000523 25357021

[57] Magill L, Robertson F, Jell G, Determining the outcomes of post-mastectomy radiation therapy delivered to the definitive implant in patients undergoing one- and two-stage implant-based breast reconstruction: A systematic review and meta-analysis. J Plast Reconstr Aesthet Surg 70 (2017): 1329–1335. 28743588 10.1016/j.bjps.2017.05.057

[58] Awadeen A, Fareed M, Elameen AM. The Impact of Postmastectomy Radiation Therapy on the Outcomes of Prepectoral Implant-Based Breast Reconstruction: A Systematic Review and Meta-Analysis. Aesthetic Plast Surg 47 (2023): 81–91. 35879475 10.1007/s00266-022-03026-y PMC9945051

[59] Clemens MW, Kronowitz SJ. Current perspectives on radiation therapy in autologous and prosthetic breast reconstruction. Gland Surg 4 (2015). 10.3978/j.issn.2227-684X.2015.04.03 PMC4461707 26161307

[60] Serra-Renom J, Munoz-Olmo J, Serra-Mestre J. Fat grafting in postmastectomy breast reconstruction with expanders and prostheses in patients who have received radiotherapy: formation of new subcutaneous tissue. J Plast Reconstr Surg 125 (2010): 12–18. 10.1097/PRS.0b013e3181c49458 20048576

[61] Quinn TT, Miller GS, Rostek M, Prosthetic breast reconstruction: indications and update. Gland Surg 5 (2016). 10.3978/j.issn.2227-684X.2015.07.01 PMC4791346 27047785

[62] Lentz R, Ng R, Higgins S, Radiation therapy and expander-implant breast reconstruction: an analysis of timing and comparison of complications. Ann Plast Surg 71 (2013): 269–273. 23788143 10.1097/SAP.0b013e3182834b63

[63] Khavanin N, Yang JH, Colakoglu S, Breast Reconstruction Trends in the Setting of Postmastectomy Radiation Therapy: Analysis of Practices among Plastic Surgeons in the United States. Plast Reconstr Surg - Glob Open 11 (2023): e4800. 36817273 10.1097/GOX.0000000000004800 PMC9937102

[64] Eriksson M, Anveden L, Celebioglu F, Radiotherapy in implant-based immediate breast reconstruction: risk factors, surgical outcomes, and patient-reported outcome measures in a large Swedish multicenter cohort. Breast Cancer Res Treat 142 (2013): 591–601. 24258257 10.1007/s10549-013-2770-0

[65] Chetta MD, Aliu O, Zhong L, Reconstruction of the Irradiated Breast: A National Claims-Based Assessment of Postoperative Morbidity. Plast Reconstr Surg 139 (2017): 783–792. 28002254 10.1097/PRS.0000000000003168 PMC5373960

[66] Heller DR, Zhuo H, Zhang Y, Surgical Outcomes of Mastectomy with Immediate Autologous Reconstruction Followed by Radiation. Ann Surg Oncol 28 (2021): 2169–2179. 32974699 10.1245/s10434-020-09122-0

[67] Khansa I, Momoh A, Patel P, Fat necrosis in autologous abdomen-based breast reconstruction: a systematic review. J Plast Reconstr Surg 131 (2013): 443–452. 10.1097/PRS.0b013e31827c6dc2 23446559

[68] Fosnot J, Jandali S, Low D, Closer to an understanding of fate: the role of vascular complications in free flap breast reconstruction. J Plast Reconstr Surg 128 (2011): 835–843. 10.1097/PRS.0b013e318218fc95 21681129

[69] Fracol M, Basta M, Nelson J, Bilateral Free Flap Breast Reconstruction After Unilateral Radiation: Comparing Intraoperative Vascular Complications and Postoperative Outcomes in Radiated Versus Nonradiated Breasts. Ann Plast Surg 76 (2016): 311–314. 26545214 10.1097/SAP.0000000000000545

[70] Baumann D, Crosby M, Selber J, Optimal timing of delayed free lower abdominal flap breast reconstruction after postmastectomy radiation therapy. J Plast Reconstr Surg 127 (2011): 1100–1106. 10.1097/PRS.0b013e3182043652 21364413

[71] Schain W, Wellisch D, Pasnau R, The sooner the better: a study of psychological factors in women undergoing immediate versus delayed breast reconstruction. Am J Psychiatry 142 (1985): 40–46. 3966585 10.1176/ajp.142.9.A40

[72] Stevens L, McGrath M, Kister S, The psychological impact of immediate breast reconstruction for women with early breast cancer. J Plast Reconstr Surg 73 (1984): 619–628. 10.1097/00006534-198404000-00018 6709743

[73] Al-Ghazal S, Sully L, Fallowfield L, The psychological impact of immediate rather than delayed breast reconstruction. Eur J Surg Oncol 26 (2000): 17–19. 10718173 10.1053/ejso.1999.0733

[74] Chevray P. Timing of breast reconstruction: immediate versus delayed. Cancer J 14 (2008): 223–229. 18677129 10.1097/PPO.0b013e3181824e37

[75] Rogers N, Allen R. Radiation effects on breast reconstruction with the deep inferior epigastric perforator flap. J Plast Reconstr Surg 109 (2002): 1925–1926. 10.1097/00006534-200205000-00022 11994594

[76] Capizzi R The preclinical basis for broad-spectrum selective cytoprotection of normal tissues from cytotoxic therapies by amifostine (Ethyol). Eur J Cancer 32 (1996): S5–S16. 10.1016/s0959-8049(96)00333-4 8976816

[77] Singh VK, Seed TM. The efficacy and safety of amifostine for the acute radiation syndrome. Expert Opin Drug Saf 18 (2019): 1077–1090. 31526195 10.1080/14740338.2019.1666104

[78] Felice PA, Nelson NS, Page EE, Amifostine Reduces Radiation-Induced Complications in a Murine Model of Expander-Based Breast Reconstruction: Plast Reconstr Surg 134 (2014): 551e–560e. 10.1097/PRS.0000000000000543 PMC4217130 25357049

[79] Pietruski P, Paskal W, Paluch Ł, The Impact of N-Acetylcysteine on Autologous Fat Graft: First-in-Human Pilot Study. Aesthetic Plast Surg 45 (2021): 2397–2405. 32221675 10.1007/s00266-020-01633-1 PMC8481185

[80] Reliene R, Pollard J, Sobol Z, N-acetyl cysteine protects against ionizing radiation-induced DNA damage but not against cell killing in yeast and mammals. Mutat Res Mol Mech Mutagen 665 (2009): 37–43. 10.1016/j.mrfmmm.2009.02.016 19427509

[81] Doctrow SR, Lopez A, Schock AM, A Synthetic Superoxide Dismutase/Catalase Mimetic EUK-207 Mitigates Radiation Dermatitis and Promotes Wound Healing in Irradiated Rat Skin. J Invest Dermatol 133 (2013): 1088–1096. 23190879 10.1038/jid.2012.410 PMC3594042

[82] Kim HJ, Kang SU, Lee YS, Protective Effects of N-acetylcysteine against Radiation-Induced Oral Mucositis In Vitro and In Vivo. Cancer Res Treat. 18 (2020). 10.4143/crt.2020.012 PMC7577823 32599978

[83] Yilmaz B, Turkcu G, Sengul E, Efficacy of N-Acetylcysteine on Wound Healing of Nasal Mucosa. J Craniofac Surg 26 (2015): 422–426. 26091056 10.1097/SCS.0000000000001880

[84] Tieu S, Charchoglyan A, Paulsen L, N-Acetylcysteine and Its Immunomodulatory Properties in Humans and Domesticated Animals. Antioxidants 12 (2023): 1867. 37891946 10.3390/antiox12101867 PMC10604897

[85] Wang F, Gao P, Guo L, Radio-protective effect and mechanism of 4-Acetamido-2,2,6,6- tetramethylpiperidin-1-oxyl in HUVEC cells. Environ Health Prev Med 22 (2017): 14. 29165102 10.1186/s12199-017-0616-9 PMC5664570

[86] Wilcox CS. Effects of tempol and redox-cycling nitroxides in models of oxidative stress. Pharmacol Ther 126 (2010): 119–145. 20153367 10.1016/j.pharmthera.2010.01.003 PMC2854323

[87] Hahn SM, Sullivan FJ, DeLuca AM, Evaluation of Tempol Radioprotection in a Murine Tumor Model. Free Radic Biol Med 22 (1997): 1211–1216. 9098095 10.1016/s0891-5849(96)00556-4

[88] Zhang L, Chen B, Tang L. Metabolic memory: Mechanisms and implications for diabetic retinopathy. Diabetes Res Clin Pract 96 (2012): 286–293. 22209677 10.1016/j.diabres.2011.12.006

[89] Spitz DR, Dornfeld KJ, Krishnan K, Oxidative Stress in Cancer Biology and Therapy. Humana Press (2012).

[90] Yan S, Brown S, Kolozsvary A, Mitigation of radiation-induced skin injury by AAV2-mediated MnSOD gene therapy. J Gene Med 10 (2008): 1012–1018. 18613255 10.1002/jgm.1226

[91] Epperly M, Bray J, Kraeger S, Prevention of late effects of irradiation lung damage by manganese superoxide dismutase gene therapy. Gene Ther 5 (1998). 10.1038/sj.gt.3300580 9578839

[92] Anderson PM, Lalla RV. Glutamine for Amelioration of Radiation and Chemotherapy Associated Mucositis during Cancer Therapy. Nutrients 12 (2020): 1675. 32512833 10.3390/nu12061675 PMC7352314

[93] Karimpour M, Hassanzadeh M, Zirak JM, Oral administration of alanyl-glutamine and glutamine improve random pattern dorsal skin flap survival in rats. Iran J Basic Med Sci 11 (2018). 10.22038/IJBMS.2018.29629.7153 PMC6118086 30186572

[94] Goswami S, Kandhare A, Zanwar AA, Oral L -glutamine administration attenuated cutaneous wound healing in Wistar rats. Int Wound J 13 (2016): 116–124. 24690128 10.1111/iwj.12246 PMC7949670

[95] Patel V, McGurk M. Use of pentoxifylline and tocopherol in radiation-induced fibrosis and fibroatrophy. Br J Oral Maxillofac Surg 55 (2017): 235–241. 28027781 10.1016/j.bjoms.2016.11.323

[96] Okunieff P, Augustine E, Hicks J, Pentoxifylline in the Treatment of Radiation-Induced Fibrosis. Radiat Oncol 22 (2024): 2207–2213. 10.1200/JCO.2004.09.101 15169810

[97] Berbée M, Fu Q, Garg S, Pentoxifylline Enhances the Radioprotective Properties of γ-Tocotrienol: Differential Effects on the Hematopoietic, Gastrointestinal and Vascular Systems. Radiat Res 175 (2011): 297–306. 21388273 10.1667/RR2399.1 PMC3115470

[98] Yang YL, Lee MTG, Lee CC, Pentoxifylline decreases post-operative intra-abdominal adhesion formation in an animal model. PeerJ 6 (2018): e5434. 30155353 10.7717/peerj.5434 PMC6110259

[99] Shindel AW, Lin G, Ning H, Pentoxifylline Attenuates Transforming Growth Factor-β1-Stimulated Collagen Deposition and Elastogenesis in Human Tunica Albuginea-Derived Fibroblasts Part 1: Impact on Extracellular Matrix. J Sex Med 7 (2010): 2077–2085. 20367772 10.1111/j.1743-6109.2010.01790.x PMC3543151

[100] Singh PK, Krishnan S. Vitamin E Analogs as Radiation Response Modifiers. Evid Based Complement Alternat Med 20 (2015): 1–16. 10.1155/2015/741301 PMC4558447 26366184

[101] Dirier A, Akmansu M, Bora H, The effect of vitamin E on acute skin reaction caused by radiotherapy. Clin Exp Dermatol 32 (2015): 571–573. 10.1111/j.1365-2230.2007.02452.x 17535282

[102] Luby A, Snider A, Mandair G, Therapeutic Interventions to Reduce Radiation Induced Dermal Injury in a Murine Model of Tissue Expander Based Breast Reconstruction. Ann Plast Surg 85 (2020): 546–552. 32187064 10.1097/SAP.0000000000002264 PMC7487044

[103] Patyar RR, Patyar S. Role of drugs in the prevention and amelioration of radiation induced toxic effects. Eur J Pharmacol 819 (2018): 207–216. 29221951 10.1016/j.ejphar.2017.12.011

[104] Obrador E, Salvador R, Villaescusa JI, Radioprotection and Radiomitigation: From the Bench to Clinical Practice. Biomedicines 8 (2020): 461. 33142986 10.3390/biomedicines8110461 PMC7692399

[105] Zhang Y, Huang Y, Li Z, Exploring Natural Products as Radioprotective Agents for Cancer Therapy: Mechanisms, Challenges, and Opportunities. Cancers 15 (2023): 3585. 37509245 10.3390/cancers15143585 PMC10377328

